# Effect of Hyaluronic Acid-Enriched Media in Embryo Implantation

**DOI:** 10.7759/cureus.27083

**Published:** 2022-07-20

**Authors:** Priti Karadbhajne, Akash More

**Affiliations:** 1 Clinical Embryology, Jawaharlal Nehru Medical College, Datta Meghe Institute of Medical Sciences, Wardha, IND; 2 Anatomy, Jawaharlal Nehru Medical College, Datta Meghe Institute of Medical Sciences, Wardha, IND

**Keywords:** embryo transfer, clinical pregnancy, implantation rate, hyaluronic acid, blastocyst

## Abstract

EmbryoGlue (Vitrolife, Sweden) is a hyaluronan-rich medium manufactured specifically for embryo transfer. Normally, culture media is used for embryo transfer. Culture media is enriched with albumin. Embryo transfer success depends upon the implantation rate. According to some researchers, hyaluronic acid-enriched media may be responsible for the success of embryo transfers. The aim of the study is to check the efficacy of hyaluronic acid-enriched media for previous in-vitro fertilization (IVF) failure patients. A 48-year patient was unable to conceive naturally for 20 years. The patient was enrolled for an IVF cycle at Wardha Test Tube Baby Centre in November 2020. The patient was enrolled for three IVF cycles. She was counseled for IVF treatment after failure of three intrauterine inseminations (IUI). Due to menopause, the patient was suggested for IVF with a donor egg and husband's sperm. The uterine septum was found in hysteroscopy and a septum section was done. The patient was conceived after the sixth embryo transfer by using EmbryoGlue. The case report highlighted that the pregnancy may be achieved by the use of EmbryoGlue for embryo transfer.

## Introduction

For the success of in-vitro fertilization (IVF), embryo implantation is an important factor. A clinical pregnancy rate is usually 40-50% for embryo implantation in IVF [1]. There are many possible reasons for the failure of embryo transfer, such as endometrium thickness, its receptivity, defects in the embryo, and hereditary factor [1]. The composition of the culture medium used for embryo transfer is a major factor in implantation. Normally, it can be used on embryos at any stage of development, including cryopreserved embryos [2]. Embryos should be in the medium for at least 10 minutes before being transferred, according to the manufacturer [2]. The effect of varied exposure times was explored in the study summarized in some studies [1-3].

It is accepted globally that the success of the embryo transfer process is hidden in the implantation technique. Most of the clinical tools used by clinicians fail to diagnose and give treatment for the reason of implantation failure [2]. Most of the evidence has found that human serum albumin (HSA) containing is not associated with success rate. So, the need was for another media to increase the success rate of implantation of the embryo. EmbryoGlue (Vitrolife, Sweden) is commercial media with a high concentration of hyaluronic acid (also known as hyaluronan) and less quantity of albumin [3]. Hyaluronan is a major macromolecule present in the female reproductive tract. It is synthesized by granulosa and cumulus cells. It is a major glycosaminoglycan present in the cervical mucus, the cumulus, follicular fluid, and seminal plasma. It is also present in the endometrium with an important role in embryo implantation. Naturally, an increase in the level of hyaluronic acid throughout the secretory phase of the menstrual cycle occurs and decreases at menstruation. CD44 receptors are present on mature oocytes and pre-implantation embryos and are also found in the human endometrium. There are some findings related to the beneficial effect of hyaluronan. It was proved beneficial for in-vitro embryo development and implantation for the first time in a mouse [3,4]. It is a linear polysaccharide chain of D-glucuronic acid and N-acetyl-D- glucosamine residues. [4].

Oocyte harvesting, embryo culture, sperm preparation, and embryo transfer are all done with commercial media nowadays. The role of ions, amino acids, and carbohydrates is emphasized in these media [3].

## Case presentation

Patient-specific information 

This case report refers to a couple who visits Wardha Test Tube Baby Centre to treat infertility in November 2019. A 48-year-old female patient with primary infertility for 20 years was enrolled for three IVF cycles. Consent was taken from the couple. Her spontaneous menses were regular after 30 days. Bleeding in menses was for four days and menstrual loss was average. The male was a daily-wage worker and the patient's occupation was of a nurse. The couple had no habit of consuming liquor, eating tobacco, or smoking on daily basis. 

Couple's medical history

This case includes a female who underwent the procedure of hysteroscopy. The consent was taken from the patient for procedures related to IVF. The uterine septum was present and bilateral ostia was seen. Septum resection was done. In a pelvic scan, the wall thickness appears normal. They had faced primary infertility, with the hope of giving birth to a baby after 19 years from 20 years of marriage. Semen analysis of husband was normal. Sperm count was 72 mil/ml and motility was 85%. The normal morphology of sperm was 9%. The husband was taking medicine for hypertension. 

They had no family history of any of these conditions, such as diabetes, hypertension, tuberculosis, asthma, seizure disorder, thyroid gland disease, or any other major diseases or surgeries. They had no previous history of psychiatric illness. The patient underwent the intrauterine insemination (IUI) treatment three times with the semen of a male partner. In IUI, a semen sample is processed with media in a centrifuge machine. Abnormal and dead sperm are washed away in this process. Only motile sperms are inserted in the uterus of females with the help of an IUI canula. All attempts of IUI failed in this patient. 

Patient's medical history

The hormone value on Day 2 of menses should be: estrogen < 50 and luteinizing hormone (LH) < 5. The thyroid value for infertility patients should be less than 2.5. Lesser or more value of thyroid in the female patient will not allow her to conceive. Sugar in blood should be investigated and more or less value of sugar in infertility patients can become a reason for the anomaly in the baby. Hormone values for patient were: estradiol - 121.28 pg/ml, LH - 0.28 mIU/ml, and thyroid-stimulating hormone (TSH) - 1.32μIU/ml. Anti-mullerian hormone (AMH) value of the patient was 0.12, which directly affects the follicle count. The value of AMH shows poor ovarian reserve in a patient. 

Due to low AMH and age factor, the patient was not able to produce the ovum to pick up retrieval. Embryos were prepared by using oocytes from the donor and sperm of the patient's husband. The patient was counseled for direct embryo transfer. Day 5 embryo was assessed and frozen for further use. During each follow up, the thickness of the endometrium is checked and observation of the gradual increase in the thickness of the endometrium is done which helps in the proper and successful implantation of the embryo in the uterus. Endometrium thickness should be less than 5 mm to start medication for embryo transfer. She started with the tablet Estrabet (E2) 2 mg twice daily and Duphaston 10 mg three times per day from Day 3 of the cycle.

Embryo thawing was done with the help of a Kitazato thawing kit (VT 601, Japan) and kept in MINC Benchtop Incubator (Cook Medical, Bloomington, USA) for two hours till embryo expansion. After two hours of thawing, two Day 5 embryos of 4AA grade were transferred. These embryos are known as blastocysts and are of good quality. The embryos were transferred without any obstacle in January 2022. This was her sixth embryo transfer. Testing of serum β-hCG was undertaken after 14 days of embryo transfer in a central clinical laboratory, Acharya Vinoba Bhave Rural Hospital (AVBRH), Sawangi, India. 

Important follow-up diagnostic and other test results

**Figure 1:**
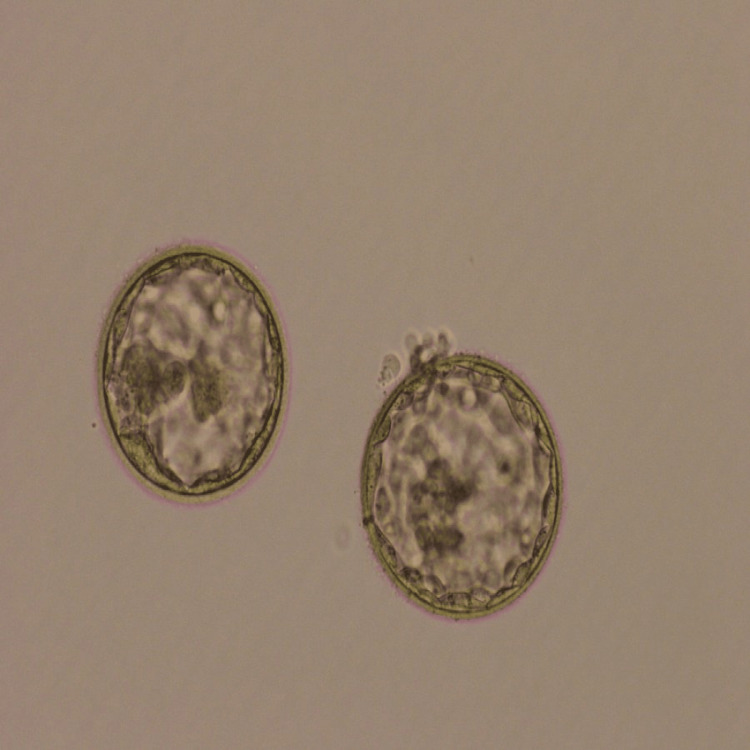
2 Day 5 Blastocyst (Grade- 4AA)

Follow-up is necessary after the process of embryo transfer. A regular follow-up is necessary for up to 14 days. A patient was discharged from the hospital after embryo transfer. Regular medication was checked for oral and injectable hormone medications. On Day 14, the blood sample was sent for the β-hCG test and showed a positive result. β-hCG value was 655 mIU/ml. Out of the two transferred embryos, only one embryo of 4AA grade was implanted (Figure 1).

## Discussion

There can be different reasons for the failure or success of embryo transfer. It can include receptivity and thickness of the endometrium, hormonal imbalance, embryo quality, embryo transfer media, technique of loading in embryo in embryo transfer catheter, etc. In our case report, the patient went through continuous failure in IVF procedures for unknown reasons. So, we tried to find the reason behind the failure in IVF procedures and changed the embryo transfer media. There are lots of reasons for failure in embryo transfer, such as the age of the patient and her husband as well as the stress that comes with continuous failure of the IVF procedure. After a particular age, the female body does not support the production of particular hormones as well as some component like hyaluronic acid. The use of hyaluronic acid-enriched media can increase the possibility of pregnancy.

The success of implantation depends on embryo competence during development and the receptiveness of uterus in a repetitive, cyclical manner during the consecutive and synchronized interactions between the embryo and endometrium [1]. Embryo implantation is successful at the blastocyst stage when the weak apposition occurs for the blastocyst, which is then attached to the surface of the endometrial epithelium, followed by trophoblast invasion through the epithelium into the endometrial stroma [2,3]. There are several reasons why embryos fail to implant despite a favorable transfer.

Embryo implantation preparation needs substantial modification of the uterine microenvironment involving an arranged coevality of complicated interactions between a viable, well-developed embryo and the hormone-dependent, receptive endometrium. Theoretically, implantation can fail due to an inappropriate embryonic function required for implantation, possibly also due to genetic inability and unsuitable uterine receptivity [3,4].

The constituents of the embryo transfer media around the embryo are a major factor for implantation. The embryo transfer media have undergone several changes to enhance implantation and pregnancy rates. Protein supplementation, the most common of which is albumin, has been frequently employed in the embryo transfer medium. Albumin is used commonly in traditional culture media [5]. Albumin is a source of energy, hormones, vitamins, and metals found in abundance in the female reproductive tract. Furthermore, albumin not only adds viscosity to the culture media but also works as a lubricant, making the embryo easier to handle and preventing embryo adhesion to the culture plate [4,5]. Serum albumin is extracted from the blood; it is an impure substance. The probability of contamination from viruses is always present with albumin. It can cause the possibility of disease transmission and biological variations. Albumin is one of these macromolecules, and it has long been the predominant macromolecule in most culture conditions for human embryo in-vitro growth [6]. Hyaluronic acid secretes naturally during the mid-proliferative phase and in the late secretory phase. It produces a viscous solution that may support the embryo transfer process and avoid exclusion from the uterine cavity [7].

Hyaluronic acid is called glycosaminoglycans (GAGs) and has distinctive characteristics that distinguish it from the other GAGs: it is non-sulfated and it is a linear polysaccharide of thousands of repeated units of alternating D-glucuronic acid and N-acetylglucosamine [7,8]. It can be used for all stages of embryo development including cryopreserved embryos. There is one more interesting feature of hyaluronan binding with water molecules that can improve tissue hydration [8]. EmbryoGlue is an embryo transfer medium with a high hyaluronan concentration (0.5 mg/mL) and a low recombinant human albumin concentration (rHA = 2.5 mg/mL). In comparison to EmbryoGlue, the standard blastocyst culture medium (Onestep) is supplemented with 10 mg/mL of rHA and a reduced dose of hyaluronan (0.125 mg/mL) [6-8].

EmbryoGlue does not contain ethylene diamine tetraacetic acid (EDTA), which is present in Onestep media. Conventional Onestep culture medium contains only non-essential amino acids, whereas EmbryoGlue contains both non-essential and essential amino acids [6]. EDTA is essential for the development of cleavage stage embryos. However, it hinders the development of blastocysts. Similarly, only non-essential amino acids stimulate cleavage stage embryo growth, whereas essential and non-essential amino acids are necessary for blastocyst development. As a result, it is assumed that EmbryoGlue is the best medium for blastocyst growth [7]. The patient's serum, fetal cord serum, commercially pooled, and more recently rHA are all sources of albumin for culture medium [8]. Hyaluronic acid has lately been recommended as a supporting macromolecule in culture media. According to some researchers, it acts as an adhesive element in embryo implantation. This macromolecule also found in the oviduct and uterine secretions, grows until implantation [8]. Hyaluronan is a polysaccharide made up of D-glucuronic acid and N-acetyl-D-glucosamine residues that alternate. The glycosaminoglycan hyaluronan is found in the cervical mucus, cumulus, follicular fluid, and seminal plasma [9,10].

When mouse embryos are cultivated and shifted in a medium with hyaluronan isolated from rooster comb or as a recombinant material, they can successfully develop and implant. An inflated concentration of hyaluronan improves in-vitro growth and the number of cells in bovine embryos by increasing the viscosity of the culture medium. They also discovered that embryos cultivated with hyaluronan supplementation had greater survival rates following freezing [1-3,10,11]. Onestep with increased hyaluronic acid concentration can be used successfully as a macromolecule in a medium used for embryo transfer which gives outcome in high pregnancy and implantation rates in a recent study [12]. EmbryoGlue medium has a high hyaluronan concentration (0.5 mg/mL) and a low albumin concentration (2.5 mg/mL). When compared to EmbryoGlue, one step includes a reduced dose of hyaluronan (0.125 mg/mL) and is supplemented with 10 mg/mL of albumin [12,13]. Adeniyi reassured in their cohort study that the hyaluronic acid-rich medium is not associated with poor results like abortion, change in gestation, etc. [1].

Previous studies stated that hyaluronic acid-enriched media does not support the adverse impact like abortion, change in gestation, multiple birth, etc. Most studies highlighted that hyaluronic acid does not act as attachment glue for embryos. Some studies revealed that the hyaluronic acid-rich media is proven as good media, at least in case of some recurrent implantation failure, and can improve the clinical pregnancy result. In our case report, a patient conceived in sixth embryo transfer with a slight change in embryo transfer media. We tried EmbryoGlue as an embryo transfer medium to check that patient could conceive and the result was seen to be positive. Here, we reported the success of patient with a change in embryo transfer media during embryo transfer. This shows that the success of embryo transfer may be achieved in a patient with previous implantation failure due to a change in embryo transfer media.

## Conclusions

This case of embryo transfer summarized the success of a patient after six failures of embryo transfer. It highlighted the use of hyaluronic acid-enriched embryo transfer media being the reason for positive clinical pregnancy. For repeated IVF embryo transfer failure patients, sequential Day 3 and Day 5 stages along with EmbryoGlue may improve clinical pregnancy.
